# Gain enhancement for wideband end-fire antenna design with artificial material

**DOI:** 10.1186/s40064-016-3039-y

**Published:** 2016-09-15

**Authors:** Min Wei, Yuanhua Sun, Xi Wu, Wu Wen

**Affiliations:** 1 The College of Computer Science, Chengdu University of Information Technology, Xuefu Road, Chengdu, 610225 China; 2 The College of Computer Science, Neijiang Normal University, Dongtong Road, Neijiang, 641100 China

**Keywords:** Wideband, End-fire antenna, Gain enhancement, Split-ring resonators, Artificial material

## Abstract

Gain enhancement wideband end-fire antenna is proposed in this paper. The proposed antenna can achieve gain enhancement by loading novel artificial materials structures (Split-ring Resonators) in the end-fire direction while broad bandwidth is realized by using elliptic dipole elements and a microstrip to coplanar balun. The measurements show that the proposed antenna have around 5–8 dB gain in the working band (5–11 GHz), which is around 2 dB more than the unloaded one. This antenna can be used in target recognition systems for its advantages of end-fire radiation broad bandwidth and high gain.

## Background

Recently, antennas are much demanded such as high gain, broad bandwidth and end-fire radiation patters with the development of military technology, especially in the field of target recognition. Meanwhile, the artificial materials because of its excellent electromagnetic characteristics have been widely used in antenna design (Caloz and Itoh 2005). Artificial materials composed by periodic structures such as split-ring resonators (Pendry et al. 1999), complementary split-ring resonators (Baena et al. 2005), composite right/left-handed transmission lines (CRLH TLs) (Lai et al. 2004) and electric LC resonators (Schurig et al. 2006). A microstrip antenna with zero-index materials(ZIM) whose permeability close to zero can achieve 1–2 dB gain improvement (Ma et al. 2009). Great interests have been focused on meta-based antennas. Among them, low/zero-index meterials (LIM/ZIM) have features of controlling the direction of emission (Lovat et al. 2007; Zhou et al. 2009). In Zhou et al. (2009), with ZIM structure as the superstrate of a microstrip patch antenna, 1–2 dB gain improvement was obtained. However, this kind of structure makes the antenna thick in profile and heavy in weight. In Huang et al. (2008), Huang proposed split-ring resonator(SRR) structure that can be used in the design of antenna. When dipole antenna is brought close to the SRR array, the antenna can achieve around 3 dB more than the gain of the dipole antenna in free space. However the bandwidth of these antennas are quite narrow.

The bowtie dipoles are widely used for their simple structure and broad bandwidth (Lin and Tsai 1998; Kiminami et al. 2004). In Qu et al. (2008), a wideband periodic endfire antenna with an impedance bandwidth of 51.4 % and unidirectional patterns was proposed based on the idea of a log-periodic antenna. Nevertheless, its gain with an average of 4 dB was still low.

In this paper, gain enhancement for wideband end-fire antenna by using split-ring resonator (SRR) structures is designed for target recognition system. The broad bandwidth of the proposed antenna is obtained by using the balun of microstrip-to-slotline transitions. Gain enhancement is achieved by loading two rows of split-ring resonator structures (SRRs) symmetrically along the end-fire direction while maintaining the wideband performance of the proposed antenna. The proposed end-fire antenna that loaded SRRs can obtain 5–8 dB gain in the whole operating band (5–11 GHz), which is around 2 dB more than unloaded one. The proposed antenna is simulated by using the high frequency structure simulator (HFSSv15) software based on the finite element method. The measured results of the fabricated antenna show good agreement with the simulated results. The proposed broadband SRRs-loaded antenna with end-fire patterns has more valuable in target recognition systems.

## Antenna design

**Fig. 1:**
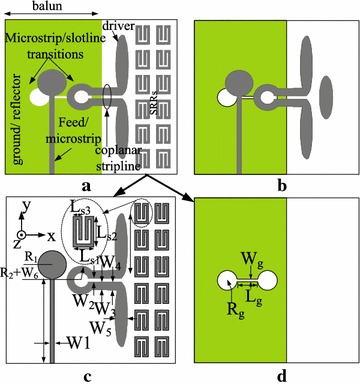
The proposed antenna **a**: overall view of proposed antenna; **b**: overall view of conventional antenna; **c**: *Top view* of the proposed antenna; **d**: *Bottom view* of the proposed antenna

**Fig. 2:**
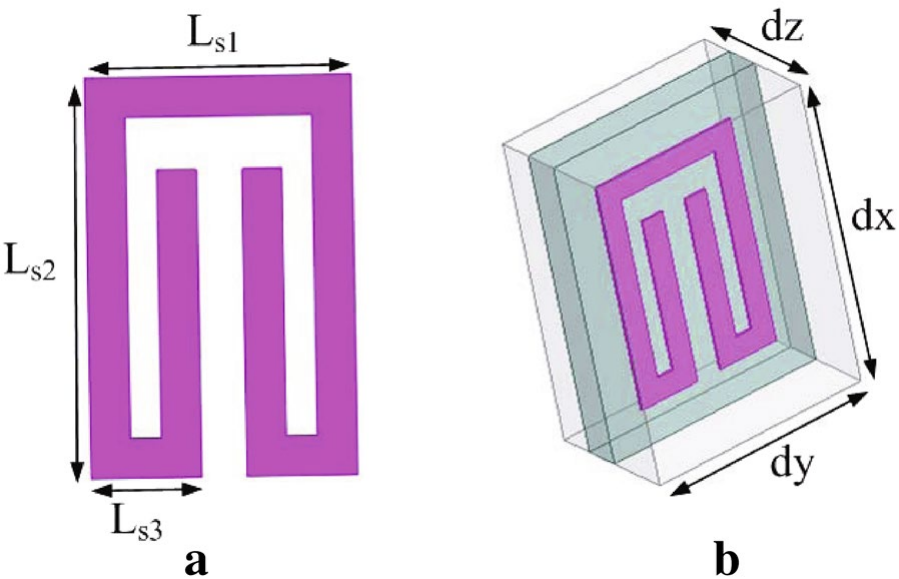
The proposed SRR unit and simulation model. **a** Details of the SRR unit cell. **b** The simulation model

The proposed wideband end-fire antenna with SRRs and conventional end-fire antenna are shown in Fig. 1. In the design of the proposed antenna, the feed uses microstrip-to-slotline transition. The proposed antenna uses the slotline at the bottom to couple the coplanar stripline at the top layer. The circular slots at the bottom layer and microstrip patch at the top layer are used to achieve the required impedance matching across the operating band(5–11 GHz). In the design of the proposed antenna, the directors of the conventional end-fire antenna are replaced by two rows split-ring resonator structures (SRRs) shown in Fig. 1. Details of the SRR unit cell are shown in Figs. 1c and 2. The split-ring resonator structure is simple and is easy to get larger capacitance. So It is convenience to tune the resonant frequency of the resonator structure by three main parameters $L_{s1}$,$L_{s2}$ and $L_{s3}$.

For demonstration purpose in the laboratory, the proposed antenna is designed on a 0.635 mm Rogers RT 6010 substrate with a dielectric constant $\varepsilon _{r}$ = 10.2. The proposed wideband end-fire antenna composes of a microstrip-to-coplanar balun, elliptic dipole elements, the metallisation on the bottom plane is a truncated microstrip ground as the reflector and eight SRRs that are printed on the top of the substrate symmetrically in the endfire direction as shown in Fig. 1. All of the proposed antenna geometric parameters are optimised using Ansoft HFSSv15. The dimensions of the proposed antenna are shown in the Table 1.

**Table 1:** The dimensions of the proposed antenna

$W_1$	$W_2$	$W_3$	$W_4$	$W_5$	$W_6$	$R_c$
1.1 mm	1.2 mm	0.6 mm	1.2 mm	1.81 mm	0.6 mm	2.1 mm

$R_1$	$L_{s1}$	$L_{s2}$	$L_{s3}$	$R_g$	$L_g$	$W_g$
1 mm	2 mm	3.1 mm	0.86 mm	1.5 mm	3 mm	0.41 mm

**Fig. 3:**
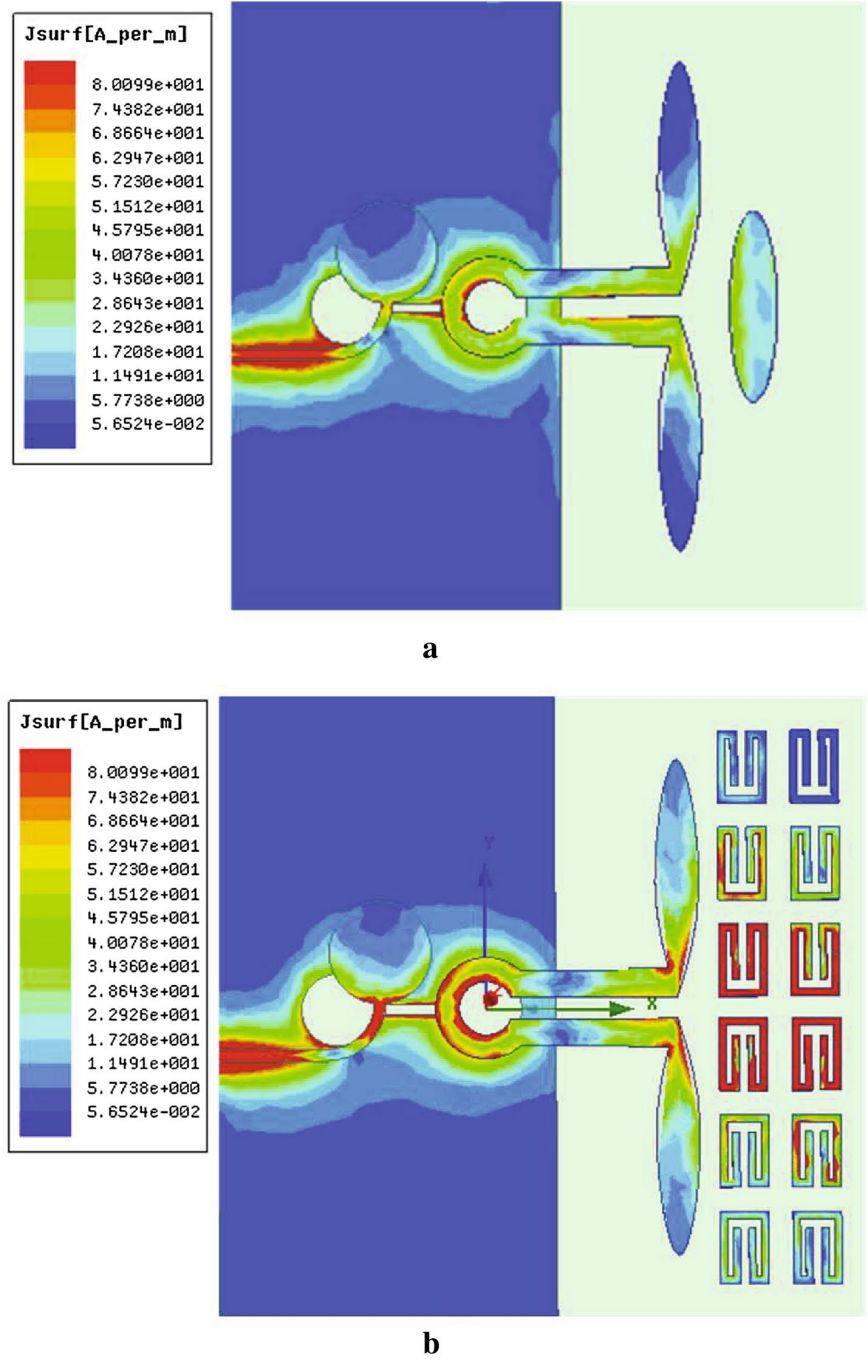
Current distribution of the two antennas; **a** antenna without loading structures; **b** antenna with SRR loading structures

**Fig. 4:**
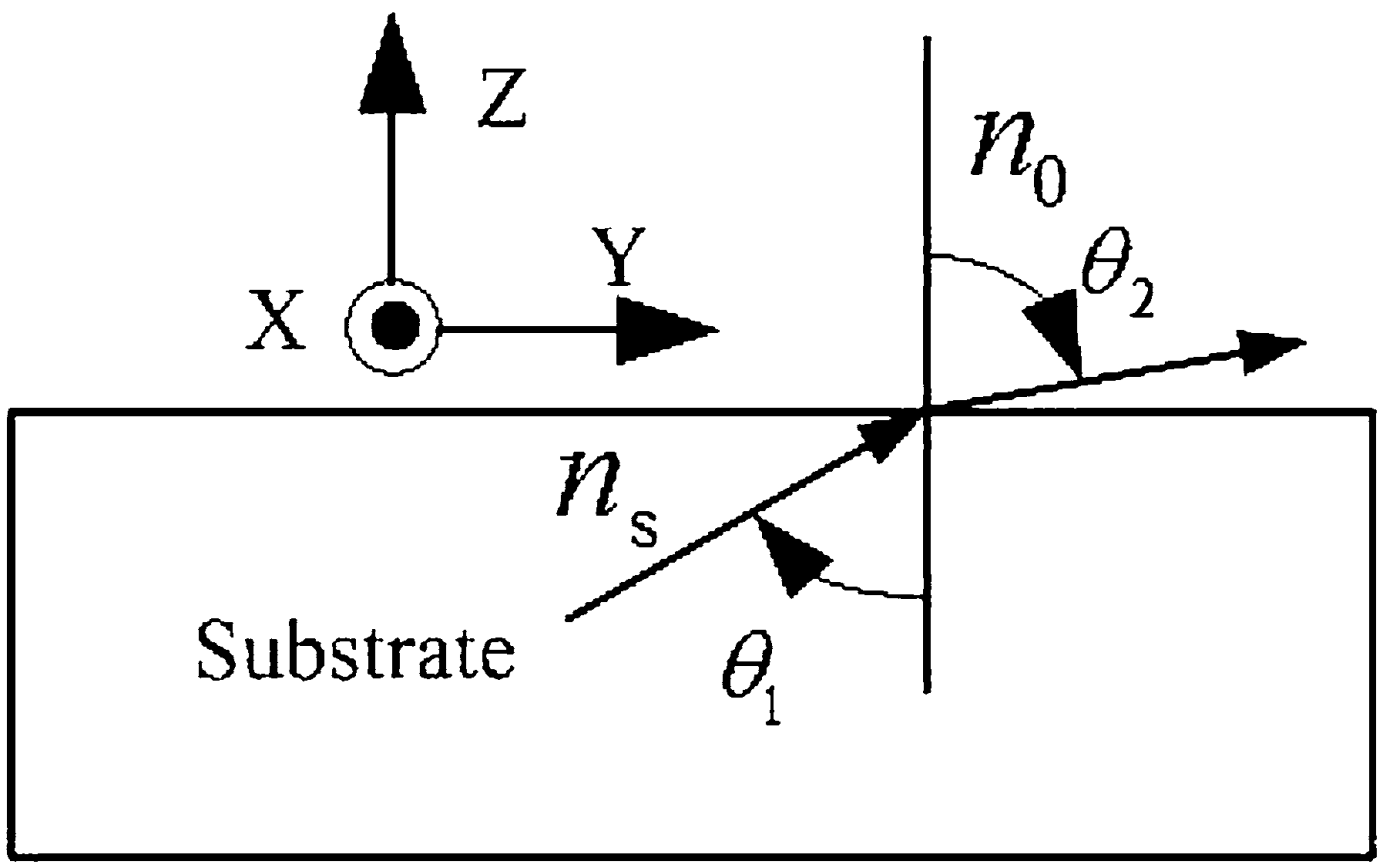
Electromagnetic wave transmitting sketch map

The SRRs loaded part consists of twelve SRRs which are printed on the surface of the substrate symmetrically in the end-fire direction. Details of the SRR unit cell are described in Fig. 2a. We use single-ring rectangular SRRs instead of dual-ring SRRs for three reasons: (a) Single-ring SRR owns a simple structure and is easy to get larger capacitance; (b) It is more convenience to turn the resonant frequency of the single-ring SRR structure by two main parameters $L_{s2}$ and $L_{s3}$; (*c*) The asymmetric structure of the inner and outer rings in the dual-ring SRR structure contributes gain enhancement for the two opposite directions. However, gain enhancement is better in one direction for the sing-ring SRRs which is a good choice for the proposed periodic end-fire antenna.

Operation principle is analyzed by investigating the current distributions of both the unloaded and loaded antennas which is given in Fig. 3. Comparing the current distributions of the SRR-loaded antenna with the unloaded one,we find that the SRR part makes vital contribution on the current movement. Further investigation on the SRR structure is made to find the relationship between gain enhancement and the characteristics of the SRR-loaded part. The most common and effective method to extract parameters is given in Chen et al. (2004), Smith et al. (2005). The scattering parameters of the model can be obtained from simulation results of HFSS, and are turned into structural parameters through methematical calculations. Figure 2b is simulation model for extracting the effective index of metamaterials. The perfect electric conductor (PEC) and perfect magnetic conductor (PMC) boundary conditions are set in the up-down faces and front-back faces of the box,respectively. The SRR can create the strongest resonance when the E-field is aligned parallel with XY-plane and the H-field is aligned with the center axis(z)of SRR. The dimension of the box are dx = 4 mm, dy = 3 mm, dz = 1.9 mm. The dimension of dx, dy of the simulation configuration which can be considered as the periodicity of the unit cell in the x- and y-directions respectively, is about the same s is given in Fig. 1 (which is depicted in dash ellipse). Lager dx or dy leads to lower resonant frequency, while larger dz leads to larger resonant frequency.

**Fig. 5:**
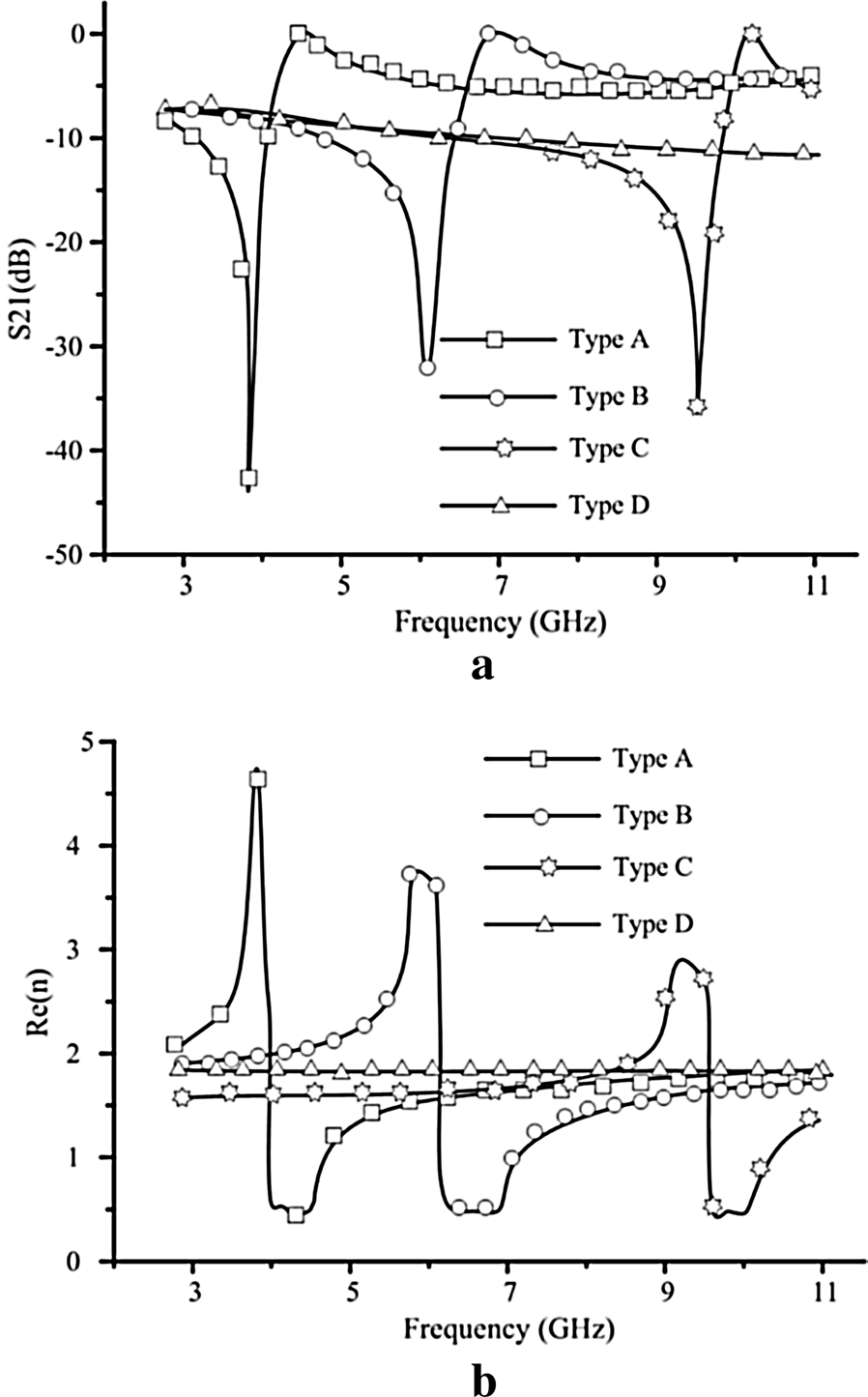
**a** Simulated S-parameters (S21) of the SRR unit. **b** Retrieved parameters from the simulated S-parameters of the SRR unit. *Type A*: $L_s1=2$ mm, $L_s2=3$ mm, $L_s3=0.83$ mm. *Type B*: $L_s1=2$ mm, $L_s2=2.5$ mm, $L_s3=0.83$ mm. *Type C*: $L_s1=2$ mm, $L_s2=3$ mm, $L_s3=0.95$ mm. *Type D*: No SRR is loaded, only substrate without copper is used

Both simulated S-parameters (S21) of the SRR unit and retrieved parameters from the simulated S-parameters of the SRR unit are presented in Fig. 5. Four type of loading structures are considered for controlling the performance of the antenna. On the one hand, from Fig. 5a, we can find that SRR metal structure affects the transmission characteristics of the whole loading unit cell. When it is working at the resonant frequency, S21 is so small that little energy can transmit through the substrate with the SRRs and thus the antenna with this loading structure own low gian at resonant frequencies. On the other hand, from Fig. 5b, we can find that when SRR unit cell resonants, the retrieved parameters (Re(n)) from the simulated S-parameters owns a sudden abrupt change. From snell' s law $n_s \cdot sin \theta _1 = n_0 \cdot sin \theta _2$, which is shown in Fig. 4. When the refractive index($n_s$) of the loading structure gets larger, with the input angle $\theta _1$ unchanged, the output angle $\theta _2$ get larger, so the energy can congregate according to endfire reference plane (xy-plane). Thus the high gain can be realized in the endfire direction. As shown in Fig. 5b, when the working band of the antenna is higher than the resonant frequency, Type A as an example, the refractive index is lower than that of the substrate (Type D) and thus gain enhancement can be obtained in the whole working band of the antenna. The same conclusion can also be deduced from the transmission characteristics of the loading unit cell which is given in Fig. 5a.

## Measurement results and discussion

**Fig. 6:**
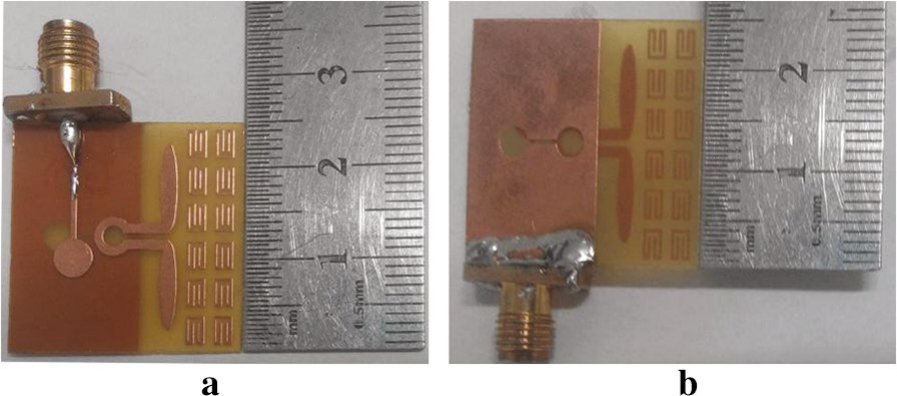
The fabricated proposed antenna **a** *Top* view; **b** *Bottom* view

**Fig. 7:**
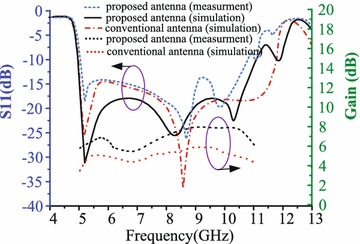
Simulation and measured return losses and gain for the proposed end-fire antenna and conventional end-fire antenna

As shown in Fig. 6, A prototype was fabricated to verify the proposed antenna design. All measured results of the proposed antenna were carried out in anechoic chamber using a vector network analyzer (VNA). All simulations were performed by Ansoft high-frequency structure simulation (HFSSv15) based on the finite-element method. As shown in Fig. 7, the measurement results are very good agreement with the simulation results. The proposed antenna can cover wide bandwidth from 5 to 11 GHz, the measurement and simulation data of the proposed antenna are in good agreement. Figure 7 shows the proposed antenna can achieve high gain from 6 to 8 dB in the operating bandwidth from 5 to 11 GHz in the end-fire direction. Figure 7 and Table 2 show the gain of the proposed antenna (loaded SRRs) have 2 dB more than the gain of the conventional antenna (unloaded SRRs) while the proposed antenna maintaining the wide bandwidth.

**Table 2:** The Gain of the proposed antenna loaded SRRs and the conventional antenna unloaded SRRs

Antenna	5 GHz (dB)	6 GHz (dB)	7 GHz (dB)	8 GHz (dB)	9 GHz (dB)	10 GHz (dB)	11 GHz (dB)
The proposed antenna	6	6.8	5.9	7	8	7.9	5.9
The conventional antenna	3.9	5	4.3	5.6	6	5.5	4.3

**Fig. 8:**
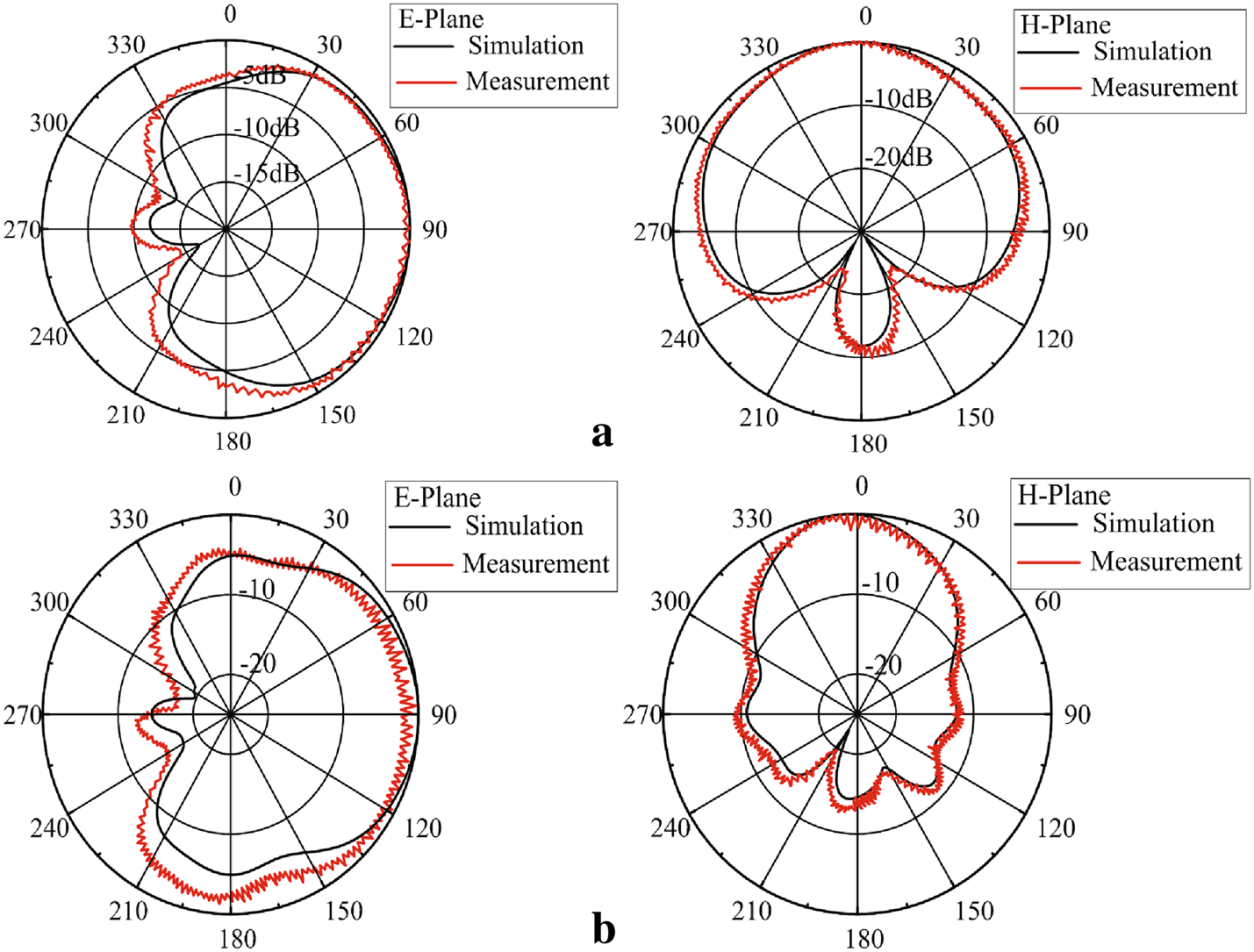
The radiaiton pattern of proposed end-fire antenna **a** 6 GHz, **b** 8 GHz

The radiation patterns of the proposed end-fire antenna are shown in Fig. 8. The measurement and simulation radiation pattern are in good agreement.It is clear that the proposed antenna has a good end-fire performance while its front-to-back ratio is better than 15 dB across the 75 % fractional bandwidth. The proposed antenna has a single main beam in the end-fire direction.

## Conclusion

In this letter, a gain enhancement wideband End-fire Antenna has been proposed. The proposed antenna uses split-ring resonators structures (SRRs) and achieves gain enhancement by using the magnetic resonators concept in the antenna design. By substituting the conventional director of the parasitic element in the conventional end-fire antenna with SRRs directions, gain enhancement is achieved in the end-fire direction while maintaining the wideband performance. The measurements show that the SRRs-loaded antenna can achieve 5–8 dB gain in the whole working band (5–11 GHz), which has a fractional bandwidth of 75 %, more than 15 dB front-to-back ratio and around 2 dB more than the gain of the conventional one that unloaded SRR structures. The proposed antenna is valuable in target Recognition systems for its advantages of broad band width,high gain and end-fire performance.
